# Comparative Structural and Antigenic Characterization of Genetically Distinct *Flavobacterium psychrophilum O*-Polysaccharides

**DOI:** 10.3389/fmicb.2019.01041

**Published:** 2019-05-08

**Authors:** John O. Cisar, C. Allen Bush, Gregory D. Wiens

**Affiliations:** ^1^United States Department of Agriculture, Agricultural Research Service, National Center for Cool and Cold Water Aquaculture, Kearneysville, WV, United States; ^2^Department of Chemistry and Biochemistry, University of Maryland, Baltimore County, Baltimore, MD, United States

**Keywords:** *Flavobacterium psychrophilum*, fish pathogen, lipopolysaccharide, *O*-polysaccharide structure, *O*-polysaccharide genes, O-serotypes

## Abstract

Little is known about the underlying basis of serotype specificity among strains of *Flavobacterium psychrophilum*, the agent of rainbow trout fry syndrome and bacterial cold-water disease. The identification of different heat-stable O-serotypes among strains of this gram-negative pathogen does, however, suggest structural variations in the *O*-polysaccharide (O-PS) moiety of cell surface lipopolysaccharide (LPS). A trisaccharide composed of L-rhamnose (L-Rha), 2-acetamido-2-deoxy-L-fucose (L-FucNAc) and 2-acetamido-4-R-2,4-dideoxy-D-quinovose (D-Qui2NAc4NR), where R represents a dihydroxyhexanamido derivative, was previously identified as the repeating unit of *Fp* CSF259-93 O-PS. Interestingly, the O-PS gene cluster of this strain and that of *Fp* 950106-1/1, which belongs to a different O-serotype, are identical except for *wzy*, which encodes the putative polymerase that links trisaccharide repeats into O-PS chains. We have now found from results of glycosyl composition analysis and high-resolution nuclear magnetic resonance, that the linkage of D-Qui2NAc4NR to L-Rha, which is α1-2 for *Fp* CSF259-93 versus β1-3 for *Fp* 950106-1/1, is the only structural difference between O-PS from these strains. The corresponding difference in O-serotype specificity was established from the reactions of rabbit and trout anti-*F. psychrophilum* antibody with purified O-PS and LPS. Moreover, LPS-based differences in antigenicity were noted between strains with O-PS loci identical to those of *Fp* CSF259-93 or *Fp* 950106-1/1, except for the genes predicted to direct synthesis of different R-groups in Qui2NAc4NR. The findings provide a framework for defining the genetic basis of O-PS structure and antigenicity and suggest that the repertoire of *F. psychrophilum* O-serotypes extends beyond what is presently recognized from serological studies of this important fish pathogen.

## Introduction

*Flavobacterium psychrophilum*, the agent of rainbow trout fry syndrome and bacterial cold-water disease, poses a serious threat to the salmonid aquaculture industry (Cipriano and Holt, 2005; Starliper, 2011). This threat is not from a single pathogenic entity but instead from several different host-specific serotypes (Mata et al., 2002; Izumi et al., 2003) and a wide range of multilocus sequence types (ST) (Nicolas et al., 2008). Accordingly, isolates of *F. psychrophilum* from diseased rainbow trout (*Oncorhynchus mykiss*) are most often identified as members of serotypes Fd or Th (Lorenzen and Olesen, 1997) and associated with STs that comprise clonal complex ST2/10 (Rochat et al., 2017) whereas other serotypes and STs are more frequently isolated from different species of salmon (Van Vliet et al., 2016) or non-salmonids such as ayu (*Plecoglossus altivelis*) (Fujiwara-Nagata et al., 2013). Different pathogen serotypes and STs are also commonly identified from the same outbreak of disease as well as from the same infected fish (Fujiwara-Nagata et al., 2013; Van Vliet et al., 2016; Ngo et al., 2017). Such outbreaks may play an important role in the ongoing evolution of *F. psychrophilum* by favoring the emergence of recombinants (Duchaud et al., 2018), such as those with altered serotypes. A genetic scheme for serotyping *F. psychrophilum* is needed to explore this possibility as well as other potential mechanisms of pathogenesis, host genetic resistance and immune protection.

The association of *F. psychrophilum* serotype specificity with heat stable O-antigens (Wakabayashi et al., 1994) suggests structural variations in the O-PS moiety of cell surface LPS. Previous structural characterization of *F. psychrophilum* (*Fp*) CSF259-93 O-PS, isolated following mild acid hydrolysis of LPS, revealed a trisaccharide repeating unit composed of L-Rha, L-FucNAc, and D-Qui2NAc4NR, where R is a 3,5-dihydroxyhexanoate derivative (MacLean et al., 2001). The corresponding locus for O-PS biosynthesis in this strain (Wiens et al., 2014) and related loci in 34 other strains of *F. psychrophilum* (Rochat et al., 2017) were tentatively identified by the presence of genes for synthesis of nucleotide-linked sugar precursors, glycosyltransferases and other proteins involved in polysaccharide biosynthesis. Importantly, those strains identified as serotype Fd or Th were also identified and distinguished by an allelic pair of genes for different putative O-antigen polymerases (Rochat et al., 2017). Variability in other O-PS genes, most notably those predicted to direct O-antigen R-group biosynthesis, was noted, however, among members of each serotype. Thus, *F. psychrophilum* serotypes Fd and Th, although clearly associated with different *wzy* alleles, were not associated with specific O-PS loci.

Synthesis of *Fp* CSF259-93 O-Ps by the well-studied Wzx/Wzy-dependent pathway (Islam and Lam, 2014) is expected to involve formation of the lipid-linked trisaccharide repeating unit on the inner surface of the cytoplasmic membrane, translocation of the saccharide moiety across the membrane by the action of a membrane-associated flippase (Wzx) and subsequent end-to-end polymerization of trisaccharide repeats by the action of a membrane-associated polymerase (Wzy). That the O-PS loci of *Fp* CSF259-93 and strains such as *Fp* 950106-1/1 (Rochat et al., 2017) are identical except for *wzy* suggests that the corresponding difference in O-PS structure is limited to the linkage between trisaccharide repeats. To test this hypothesis, we determined the structures of O-PS from these strains and antigenically compared each O-PS and LPS. In addition, we antigenically compared LPS from strains with O-PS loci that were either genetically identical to those of *Fp* 950106-1/1 or *Fp* CSF259-93 or non-identical with respect to the putative genes for synthesis of different R-groups in Qui2NAc4NR. The results support the notion that each genetically distinct O-PS locus is associated with a different LPS serotype.

**Table 1:** *Flavobacterium psychrophilum* strains.

Strain	Source	Accession no.	References
950106-1/1	Rainbow trout, Denmark	MK214915	Castillo et al., 2015
CSF259-93	Rainbow trout, Idaho	MK214917	Wiens et al., 2014
11754	Rainbow trout, South Dakota	MK214916	Neiger et al., 2016
Loa-10	Rainbow trout, Utah	MK095937	This study
CSF117-10	Rainbow trout, Idaho	MK095936	This study
ARS-060-14	Rainbow trout, North Carolina	MK095938	This study

## Materials and Methods

### Bacteria

The strains of *F. psychrophilum* used in the present study (Table 1) were stored as frozen stocks, cultured on plates of tryptone yeast extract salts (TYES) agar for 5 days at 15°C and handled using Biosafety Level 2 procedures as approved by the USDA/ARS North Atlantic Area Institutional Biosafety Committee. Genomic DNA was isolated using a cetyltrimethylammonium bromide (CTAB)/phenol-chloroform/ isoamyl alcohol procedure as described (Wilson, 2001) with minor modifications. Modifications included a final concentration of 170 μg/ml proteinase K during cell lysis and an additional RNase A treatment prior to final isopropanol precipitation of nucleic acids. Draft genome sequencing (∼90× coverage) was performed by The Sequencing Center (Fort Collins, CO, United States). DNA libraries were prepared using the Nextera XT Library Kit and sequenced using the MiniSeq System (Illumina) and the MiniSeq Reagent Kit, Mid Output (2 × 150 = 300 cycles, pair-end reads). Sequence data were assembled using default *de novo* assembly settings in Geneious software (v 11.1.2, Biomatters Ltd., New Zealand). GenBank accession numbers of annotated O-PS loci are listed in Table 1.

### Lipopolysaccharide Preparation

Bacteria (8–16 gm wet weight) were harvested from TYES plates, washed twice by centrifugation in 20 mM Tris-Cl buffer (pH 7.5) containing 20 mm MgCl_2_, suspended to 20% (weight/vol.), and stored at −20°C prior to phenol-water extraction. Suspensions were thawed, supplemented with 100 μg/ml Proteinase K (Epicenter), 80 μg/ml RNase A (Qiagen), and 6 units/ml DNase I (Thermo Scientific), immediately disrupted using a Branson Sonifier Cell Disruptor 350 and incubated 1 h at 50°C to allow enzyme digestion. Digests were brought to 70°C and mixed with an equal volume of saturated phenol solution (pH 6.6) at the same temperature. The mixture was stirred vigorously for approximately 12 min, cooled on ice to 10°C and centrifuged to separate the phases, which were recovered and dialyzed against tap water followed by deionized water. The dialyzed phenol phase, which contained LPS as previously noted (LaFrentz et al., 2007), was concentrated by ultrafiltration above an Ultracel^®^ 100 kDa membrane (EMD Millipore) and subjected to a second cycle of proteinase K and RNase A digestion, phenol-water extraction, dialysis and ultrafiltration as described above. Yields of phenol-phase LPS from *Fp* 950106-1/1 and *Fp* CSF259-93 were approximately 3 and 6 mg, respectively, per gram wet cells. Phenol phase LPS was hydrolyzed as described below to prepare O-PS or was further purified by HIC, which was performed following previously described protocols (Muck et al., 1999; Ren et al., 2012). For HIC, approximately 8 mg phenol-phase LPS was applied to columns (2.5 cm × 28 cm) of Butyl Sepharose 4 Fast Flow (GE Healthcare) in 0.7 M sodium acetate buffer (pH 4.5). Columns were rinsed with two column volumes of 0.7 M sodium acetate buffer to remove contaminating nucleic acid, two column volumes of 10% n-propanol to remove sodium acetate buffer, two column volumes of 30% n-propanol to elute bound LPS and two column volumes of water to remove n-propanol. The 30% n-propanol and water eluates were pooled, concentrated above a 100 kDa membrane, dialyzed against deionized water and stored at 4°C in the presence of a small drop of chloroform, which was added as preservative.

### *O*-Polysaccharide Preparation

Phenol-phase LPS (approximately 20 mg) was hydrolyzed as previously described (MacLean et al., 2001) in 6% glacial acetic acid for 2 h at 100°C to cleave O-PS from core-lipid A (Shaw, 1993). The hydrolysate was frozen at −78°C, thawed, and centrifuged (30 min × 20,000 *g*) at 4°C to pellet insoluble core-lipid A. The O-PS-containing supernatant was harvested, brought to pH 4.5 by addition of 3 M NaHAC buffer to a final concentration of 0.7 M and applied to a column (2.5 cm × 14 cm) of Butyl Sepharose 4 Fast Flow. O-PS passed through the column in 0.7 M NaHAC buffer and was recovered, concentrated to 6 ml above an Ultracel^®^ 10 kDa Ultrafiltration membrane (EMD Millipore) and applied to a column (2.5 cm × 66 cm) of Sephacryl S-100 High Resolution (GE Healthcare) equilibrated with 20 mM Tris-buffered (pH 7.6) saline containing 0.02% sodium azide. O-PS emerged near the void volume of the column and was recovered, concentrated by ultrafiltration above a 10 kDa membrane, dialyzed against water to remove salt and lyophilized.

### Biochemical Methods

The phenol-sulfuric acid assay (Masuko et al., 2005) for total carbohydrate was performed with L-rhamnose as standard. Values from this assay, which does not detect N-acetylated sugars, accounted for approximately 30% of the dry weight of purified LPS. Glycosyl composition analysis of O-PS samples (100–200 μg) was performed at the University of Georgia, Complex Carbohydrate Research Center by combined gas chromatography/mass spectrometry of the alditol acetates as described (Pena et al., 2012). Dried sample containing 20 μg of inositol as internal standard was hydrolyzed in 2 M trifluoroacetic acid (TFA) for 2 h in a sealed tube at 121°C, reduced with NaBD_4_, and acetylated using acetic anhydride/TFA. The resulting alditol acetates were analyzed on an Agilent 7890A GC interfaced to a 5975C MSD, electron impact ionization mode. Separation was performed on a 30 m Supelco SP-2331 bonded phase fused silica capillary column. A standard set of sugars was run to quantify detected monosaccharides. In addition, a sample of *Pseudomonas* sp. LPS containing bacillosamine was included to accurately identify the retention time of this residue.

### NMR Spectroscopy

Nuclear magnetic resonance was performed following methods adapted from those used in studies of pneumococcal polysaccharides (Geno et al., 2017). O-PS samples (6 mg) were lyophilized twice from 99.8% D_2_O and dissolved in 99.996% D_2_O. NMR spectra were recorded at 55°C in Bruker Advance spectrometers running Topspin 3 software at 500 and 600 MHz. All proton and carbon chemical shifts were referenced relative to internal acetone using δ ^1^H = 2.225 ppm and δ ^13^C = 31.07 ppm. Multiplicity-edited HSQC was used to distinguish methylene from methine groups. The common homonuclear two-dimensional NMR methods of DQF-COSY, TOCSY and NOESY were augmented by the hybrid method HSQC-TOCSY, which was enhanced in the crowded carbohydrate spectra by high digital resolution in the indirect dimension (^13^C). HMBC and HSQMBC spectra were used to identify linkage positions and for residue assignments. All NMR data were processed by NMRpipe and NMRDraw (NMRScience) with analysis by NMRview (One Moon Scientific).

### Rabbit and Trout Anti-*F. psychrophilum* Sera

*Flavobacterium psychrophilum* CSF259-93 or 950106-1/1 cells were harvested from TYES plates, washed three times with PBS, suspended in buffer, adjusted to an optical density of approximately 1.2 at 525 nm and stored at 4°C for a few days in the presence of 0.5% formalin, prepared by dilution of 10% Neutral Buffered Formalin (Thermo Fisher Scientific). Cell suspensions were shipped to Pacific Immunology for immunization of two rabbits per immunogen following the 13-week Antibody Production Protocol. Each animal received four subcutaneous injections of immunogen (0.25 ml for primary immunization followed by three 0.15 ml boosters) administered with an equal volume of Freund’s incomplete adjuvant. Rabbit antisera selected for study were those that gave the strongest cross reactions with heterologous LPS in immunodiffusion.

Year class 2017 ARS-Fp-R line rainbow trout (Leeds et al., 2010; Wiens et al., 2013) were maintained at the NCCCWA following Institutional Animal Care and Use Committee (Leetown, WV, United States) protocols (#98 and #139). Twenty fish per group, as part of a larger study, were immunized by intraperitoneal injection of 0.1 ml containing 80 μl immunogen (i.e., the washed *Fp* CSF259-93 or *Fp* 950106-1/1 cell suspensions described above) or PBS administered with 20% EMULSIGEN^®^ (MVP adjuvants) as recommended by the manufacture. Each fish (mean body weight 162 g) received a single injection and was bled 33 days later (422 temperature degree days). Studies were conducted with pooled antisera against each immunogen prepared from the 20 fish in each group.

### Immunological Methods

Dot blots were prepared by spotting serial dilutions of LPS or O-PS samples (1 μl/spot) on 0.45 μ HATF08250 membranes. Membranes were dried overnight, blocked 1 h in 20 mM Tris buffered saline (pH 7.5) containing 0.1% Tween 20 and 2% non-fat dry milk (blocking buffer), incubated 1 h with dilute (generally 1/15,000) rabbit anti-*F. psychrophilum* antiserum and 1 h with peroxidase-conjugated goat anti-rabbit Ig (Bio-Rad) prior to development using a Pierce DAB Substrate Kit (Thermo Fisher Scientific). The approximate sensitivity of this assay was 2 ng/ml for *F. psychrophilum* LPS and 25 ng/ml for O-PS. Immunodiffusion was performed in 1% agarose gel cast on GelBond^®^Film (Lonza) in sodium barbital buffered saline (pH 7.4) containing 0.1% Tween 20. Immunodiffusion wells were filled with rabbit anti-*Fp* 950106-1/1 antiserum, anti-*Fp* CSF259-93 antiserum that was concentrated fourfold using a Microcon-10 kDa Centrifugal Filter Unit (EMD Millipore) or solutions of O-PS or LPS at approximately 0.5 mg/ml. Gels were incubated overnight at 4°C to allow immunoprecipitation, soaked in buffer followed by water to remove soluble protein and salt, dried and stained (Crowle and Cline, 1977). Western blots were prepared from 12% acrylamide gels in pH 8.3 Tris glycine buffer containing 0.1% SDS that were transferred to nitrocellulose in Tris-glycine transfer buffer (pH 8.3) containing 20% methanol. Transfers were blocked overnight at 4°C in KPL Milk Diluent/Blocking solution (SeraCare), incubated 1 h with equivalent dilutions of rabbit anti-*Fp* CSF259-93 or anti-*Fp* 950106-1/1 antisera followed by 1 h with peroxidase-conjugated goat anti-rabbit Ig (Bio-Rad) prior to development with a Pierce DAB Substrate Kit.

ELISA was performed with Costar 3590 ELISA plates (Corning) coated overnight at 4°C with 50 ng/ml *Fp* CSF259-93 LPS or *Fp* 950106-1/1 LPS in Carbonate-Bicarbonate coating buffer (Sigma). LPS-coated plates were washed three times with TBS containing 0.1% Tween 20 (TBS-T20) immediately prior to use. ELISA inhibition reactions were set up in TBS containing 0.5% BSA (TBS-BSA) in U-bottom plates (120 μl/well) by adding rabbit anti-*Fp* CSF259-93 or anti-*Fp* 950106-1/1 antiserum, at a dilution sufficient for an OD_450_ of approximately 1.5 in ELISA, to serial twofold dilutions of LPS inhibitors. Dilutions of inhibitor were prepared from stock solutions of LPS at concentrations that ranged from 100 to 500 μg/ml, as determined from results of at least three phenol sulfuric acid assays (see above). Control reaction mixtures containing antibody alone or no antibody (for 0 and 100% inhibition of ELISA, respectively) were also included. Following overnight incubation at 4°C, reaction and control mixtures were transferred (100 μl/well) to a washed LPS-coated ELISA plate for 1 h incubation at room temperature. The ELISA plate was washed with TBS-T20, incubated 1 h with peroxidase-conjugated goat anti-rabbit IgG (Bio-Rad) in TBS-BSA, washed and developed 30 min with KPL SureBlue TMB peroxidase substrate (SeraCare). Measurements of OD_450_ were made with an Epoch 2 Microplate reader (BioTec). Concentrations of LPS for 50% inhibition of ELISA ± standard errors were estimated from results of at least two independent experiments.

ELISA inhibition of rainbow trout antibody binding was performed as described above by incubating LPS-coated ELISA plates 1 h with reaction mixtures setup with equivalent dilutions (approximately 1/2500) of trout anti-*Fp* 950106-1/1 or anti-*Fp* CSF259-93 antiserum and decreasing amounts of LPS, 1 h with 0.1 μg/ml Warr’s 1–14 monoclonal antibody (DeLuca et al., 1983) against trout IgM and 1 h with peroxidase-labeled, affinity purified goat anti-mouse IgG (H+L) (KPL) prior to development with SureBlue TMB peroxidase substrate.

## Results

### Purification of *F. psychrophilum* LPS and O-PS

Following phenol-water extraction of *F. psychrophilum* cell lysates, virtually all antigenic activity (>95%) detected by dot immunoblotting was associated with LPS recovered from the dialyzed phenol phase. Contaminating nucleic acids, which accounted for 3–25% of the carbohydrate in LPS solutions, were readily removed by HIC performed with columns of Butyl-Sepharose. The capacity of such columns was, however, relatively low (i.e., approximately 50 μg *F. psychrophilum* LPS/ml gel), which prompted the use of phenol phase LPS, rather than HIC-purified LPS, as starting material for the preparation of O-PS. This shortcut proved useful as mild acid hydrolysis to cleave O-PS from core-Lipid A also cleaved acid-labile phosphodiester bonds, thereby allowing complete removal of contaminating nucleic acid during subsequent purification of O-PS. Starting with approximately 20 mg of phenol phase LPS, we obtained 8 mg *Fp* 950106-1/1 O-PS and 9 mg *Fp* CSF259-93 O-PS.

### Structural Characterization of *F. psychrophilum* O-PS

The results from glycosyl composition analysis of *Fp* CSF259-93 O-PS and *Fp* 960106-1/1 O-PS were essentially identical. Each O-PS yielded prominent peaks of rhamnose and N-acetylfucosamine, in roughly equal proportions, along with a very small peak of bacillosamine (Supplementary Figure S1). Speculating that the low recovery of bacillosamine from Qui2NAc4NR, which was expected from previous studies of *Fp* CSF259-93 O-PS (MacLean et al., 2001), could be due to failure of amide hydrolysis, we attempted stronger acidic hydrolysis (6 N HCl for 4 h at 100°C) of O-PS samples as well as saponification with 4 M KOH for 4 h. Neither treatment improved the yield of bacillosamine. The presence of Qui2NAc4NR in both *Fp* CSF259-93 and *Fp* 960106 O-PS was, however, clearly established by NMR as described below.

**FIGURE 1:**
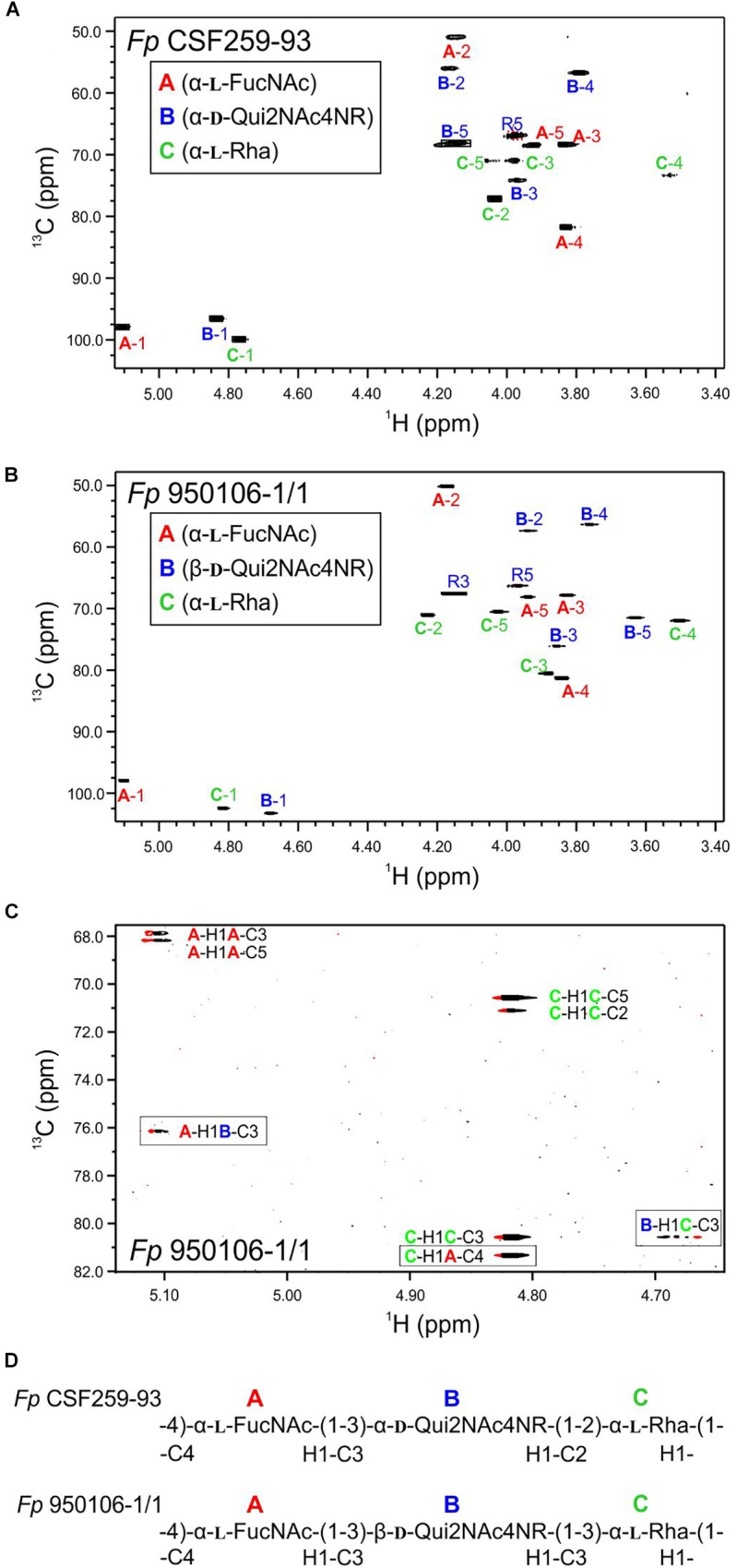
Multiplicity edited ^1^H-^13^C HSQC spectra recorded at 55°C: **(A)** *Fp* CSF259-93 O-PS; **(B)** *Fp* 950106-1/1 O-PS. **(C)** Phase-sensitive HSQMBC spectrum of *Fp* 950106-1/1 O-PS showing both intra- and inter-residue ^1^H-^13^C cross peaks; inter-residue cross peaks are identified by rectangles. **(D)** Structure of each O-PS deduced from ^1^H-^13^C HSQC and HMBC or phase-sensitive HSQMBC spectra.

The ^1^H-^13^C HSQC spectrum of *Fp* CSF259-93 O-PS (Figure 1A) was independently assigned using the same letter designations (A, B, and C) for the three sugar residues as those used previously (MacLean et al., 2001). Starting from each anomeric C-H resonance, we used scalar coupling correlation by DQF-COSY to locate each H2 and HSQC for each C2. Using HSQC-TOCSY we identified the resonances of atoms 2, 3, 4, and 5 in combination with long-range ^1^H-^13^C scalar coupling by HMBC. Given the assignment of the C-H resonances of atoms 1 through 5 of each residue, the methyl groups of the 6-deoxy sugars were assigned by HSQC-TOCSY. Despite small differences ^1^H-^13^C chemical shifts, which presumably result from small differences in temperature and chemical shift referencing, our NMR assignment for *Fp* CSF259-93 O-PS (Table 1) is in complete agreement with the assignment of MacLean et al. (2001). Likewise, the previously determined glycosidic linkage positions of *Fp* CSF259-93 O-PS are the same as those determined by long-range ^1^H-^13^C coupling correlations in the present study.

As noted for *Fp* CSF259-93 O-PS, the ^1^H-^13^C HSQC spectrum of *Fp* 950106-1/1 O-PS (Figure 1B) contained three peaks in the anomeric region, between 5.2 and 4.7 ppm in ^1^H and 103 to 97 ppm in ^13^C, thereby indicating the presence of three sugar residues in each polysaccharide. Although not shown in Figure 1, three peaks indicative of methyl groups of 6-deoxy sugars were also present in the region of 1.3–1.2 ppm in ^1^H and 16–18 ppm in ^13^C of both polymers (Table 1 and Supplementary Figure S2). Despite these similarities, differences between the spectra indicated two distinct structures.

To determine the distinct structure of *Fp* 950106-1/1 O-PS, we arbitrarily assigned the letters A, B, and C to the anomeric signals of this polysaccharide (Figure 1B). The anomeric signal of residue A at 5.106 and 97.79 ppm was correlated with A-H2 at 4.176 ppm with J_H−1,H−2_ = 3 Hz, which along with ^1^J_C−H_ = 174 Hz indicates and α-configuration for residue A. A-H2 was also correlated with A-C2 at 50.20 ppm, a chemical shift typical of a 2-acetamido sugar. The assignments of A-3, A-4, and A-5 were identified by HSQC-TOCSY correlation with A-H1 and by long range C-H correlation with A-C3 and A-C5 (Figure 1C). The NMR data indicate that residue A of *Fp* 950106-1/1 O-PS, like that of *Fp* CSF259-93 O-PS, is α-FucNAc. Moreover, the signals from this residue in Figure 1A,B, which are labeled in red, were similar for the two polysaccharides.

A sharp anomeric resonance of residue C at 4.818 ppm in ^1^H and 102.48 ppm in ^13^C were correlated by DQF-COSY (Supplementary Figure S3) with C-H2 at 4.232 ppm, which in turn was correlated with C-C2 at 71.07 ppm. HSQC-TOCSY (Supplementary Figure S2) from C-H1 showed cross peaks with C-C2 and a very weak cross peak with C-C3 at 80.57 ppm. The DQF-COSY cross peak with C-H2 showed a small homonuclear coupling to C-H3 at 3.885 ppm, indicating that residue C is the rhamnose residue detected in the glycosyl composition analysis. HSQC-TOCSY from C-C3 and DQF-COSY identify C-H4 at 3.503 ppm and C-H5 at 4.027 ppm. The α-anomeric configuration of residue C (Rha) was indicated by ^1^J_C−H_ = 169 Hz in coupled HSQC-TOCSY.

The anomeric resonance B-1 at 4.679 ppm in ^1^H and 103.28 in ^13^C shows a large homonuclear splitting (J_H−1,H−2_ = 7.5 Hz) and ^1^J_CH_ = 162.5 Hz. These data both show that, in contrast to the case of *Fp* CSF259-93, Residue B in *Fp* 150106-1/1 O-PS has a β-anomeric configuration. B-H1 in the NMR spectrum of *Fp* 950106-1/1 O-PS was correlated with B-H2 at 3.939 ppm and HSQC showed that B-C2 is at 57.37 ppm for this acetamido sugar residue, as expected for Qui2NAc4NR, the third residue of *Fp* 950106-1/1 O-PS. The axial configuration of all the ring protons leads to HSQC-TOCSY correlation of B-H1 not only with B-C2 but also with B-C3 (76.14 ppm), B-C4 (56.34 ppm) and B-C5 (71.52 ppm). The corresponding ^1^H chemical shifts were assigned by DQF-COSY and HSQC. The resonances of all the 6-deoxy sugars were assigned by HSQC-TOCSY cross peaks between the well resolved ^13^C6 resonances with the ^1^H resonances of the sugar rings.

In addition to the assignment of the NMR signals of the sugar residues, we assigned the resonances of the 3,5-dihydroxyhexanoyl R-group in O-PS of both *Fp* CSF259-93 and *Fp* 950106-1/1. The assignment of R-group NMR spectra began at the terminal methyl group, R-6 at 1.212 ppm in ^1^H and 22.93 ppm in ^13^C. HSQC-TOCSY showed correlation between R-H6 and R-C5 at 66.34 ppm. We further observed HSQC-TOCSY correlation of R-C5 to the methylene group R-4 having R-H4 at 1.617 ppm and R-H4’ at 1.738 ppm. R-C4 was located by HSQC at 45.98 ppm. HSQC-TOCSY correlated R-H4 and 4′ to R-C3 at 67.60 ppm and DQF-COSY showed the correlation of R-H3, R-H4 and R-H4′. HSQC-TOCSY identified R-C2 at 44.83 ppm with R-H2 at 2.415 ppm.

To establish the amide linkage of the 3,5-dihydroxyhexanoyl group to the O-PS, we first assigned the amide linkages of the two N-Acetyl amino sugars to residues A and B. The acetyl methyl groups appear at 1.963 ppm in ^1^H and 23.01 ppm in ^13^C and at 2.053 ppm in ^1^H and 23.14 ppm in ^13^C. We used the carbonyl-selective HMBC experiment employing a soft ^13^C pulse in the 175 ppm region to detect 3-bond long-range ^1^H-^13^C coupling correlation between amide methyl protons and the carbonyl ^13^C atoms of the amide linkage. A cross peak observed between 2.059 ppm and 175.24 ppm was accompanied by a second cross peak at 4.176 ppm corresponding to H2 of residue A (α-FucNAc). A second cross peak seen between 1.963 ppm and 174.93 ppm had a further correlation to 3.934 ppm recognized as H2 of residue B (Qui2NAc4NR). There was a third group of cross peaks in carbonyl-selective HMBC at 175.01 ppm and 2.419 ppm, the chemical shift of the R-H2 protons of the hexanoyl amide linkage to the O-PS as well as to 3.764 ppm, which corresponds to B-H4, the 4N linkage position of the hexanoyl group in Qui2NAc4NR.

**Table 2:** Residue by residue comparison of HSQC ^1^H and ^13^C chemical shifts of *Fp* CSF259-95 and *Fp* 950106-1/1 O-PS.

O-PS	Residue	Structure	Chemical shifts (ppm)					
			H-1/C-1	H-2/C-2	H-3/C-3	H-4/C-4	H-5/C-5	H-6/C-6
*Fp* CSF259-93	A	-4)-α-L-FucNAc-(1-	5.113	4.131	3.820	3.825	3.917	1.214
			97.79	50.20	67.81	81.36	67.92	16.84
*Fp* 950106-1/1		-4)-α-L-FucNAc-(1-	5.106	4.176	3.825	3.844	3.947	1.243
			97.79	50.20	67.81	81.36	67.92	16.84
*Fp* CSF259-93	B	-3)-α-D-Qui2NAcR-(1-	4.838	4.160	3.957	3.786	4.147	1.207
			96.38	55.35	73.69	56.07	67.73	17.67
*Fp* 950106-1/1		-3)-β-D-Qui2NAcR-(1-	4.679	3.939	3.858	3.764	3.630	1.248
			103.28	57.38	76.14	56.34	71.52	18.05
*Fp* CSF259-93	C	-2)-α-L-Rha-(1-	4.768	4.036	3.975	3.525	4.062	1.247
			99.77	76.72	70.48	72.87	70.48	17.46
*Fp* 950106-1/1		-3)-α-L-Rha-(1-	4.818	4.232	3.885	3.503	4.027	1.214
			102.48	71.07	80.57	72.01	70.55	17.41
*Fp* CSF259-93	R	3,5-dihydroxyhexanoyl		2.419	4.157	1.622,	3.969	1.212
				44.86	67.61	1.738^∗^ 46.01	66.34	22.91
*Fp* 950106-1/1		3,5-dihydroxyhexanoyl		2.419	4.157	1.617,	3.969	1.212
				44.83	67.60	1.738^∗^ 45.98	66.34	22.93

*^∗^Values separated by a comma are H,H′ chemical shifts.*

Given the complete ^1^H and ^13^C assignments of each sugar residue (Table 2), saccharide linkages were readily determined from ^1^H-^13^C long range coupling correlations from phase-sensitive HSQMBC spectra. In the spectrum of *Fp* 950106 1/1 O-PS (Figure 1C), sugar linkage positions were indicated by a cross peak between A-H1 and B-C3, between B-H1 and C-C3 and between C-H1 and A-C4. In agreement with previous results of MacLean et al. (2001), the corresponding cross peaks in a long-range ^1^H-^13^C correlation spectrum of *Fp* CSF259-93 were between A-H1 and B-C3, between B-H1 and C-C2 and between C-H1 and A-C4 (Figure 1D). These findings indicate that the O-PS structures of *Fp* CSF259-93 and *Fp* 960106-1/1 are identical except for the linkage of D-Qui2NAc4NR to L-Rha, which is α1-2 for the former as opposed to β1-3 for the latter polysaccharide (Figure 1D).

### Antigenic Characterization of *F. psychrophilum* O-PS and LPS

**FIGURE 2:**
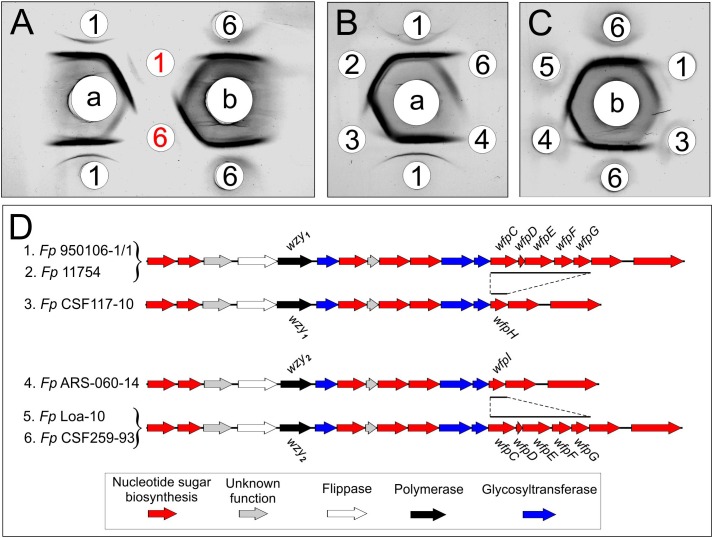
Immunodiffusion of *Fp* O-antigens: **(A)** comparison of O-PS and LPS of *Fp* 950106-1/1 and *Fp* CSF259-93; **(B)** comparison of LPS of *Fp* 950106-1/1 with LPS of other *Fp* strains; **(C)** comparison of LPS of *Fp* CSF259-93 with LPS of other *Fp* strains. Center wells contained (a) anti-*Fp* 950106-1/1 serum or (b) anti-*Fp* CSF259-93 serum. Outer wells contained (1) *Fp* 950106-1/1 LPS, (red 1) *Fp* 950106-1/1 O-PS, (2) *Fp* 11754 LPS, (3) *Fp* CSF117-10 LPS, (4) *Fp* ARS-060-14 LPS, (5) *Fp* Loa-10 LPS, (6) *Fp* CSF259-93 LPS or (red 6) *Fp* CSF259-93 O-PS. Genes that distinguish the O-PS loci of different *Fp* strains are labeled in panel **(D)**.

Immunodiffusion experiments performed with rabbit antisera against *Fp* 960106-1/1 and *Fp* CSF259-93 (Figure 2, wells a or b, respectively) indicated partial antigenic identity between O-PS from these strains (Figure 2A, red numbers) and complete identity between each O-PS and corresponding LPS (Figure 2A, black numbers). In similar experiments, reactions of complete identity were noted between LPS from strains with identical O-PS gene clusters (Figure 2B, wells 1 and 2; Figure 2C, wells 5 and 6) and partial identity between LPS from strains that contained the same *wzy* (i.e., *wzy*_1_ or *wzy*_2_) but different putative R-group genes (Figure 2B, compare well 3 to wells 1 and 2, Figure 2C, compare well 4 to wells 5 and 6). In one case, partial identity was also noted between homologous and heterologous LPS from strains that differed in both *wzy* and R-group genes (Figure 2C, well 3) while in the other case, the heterologous LPS failed to cross react (Figure 2B, well 4).

**FIGURE 3:**
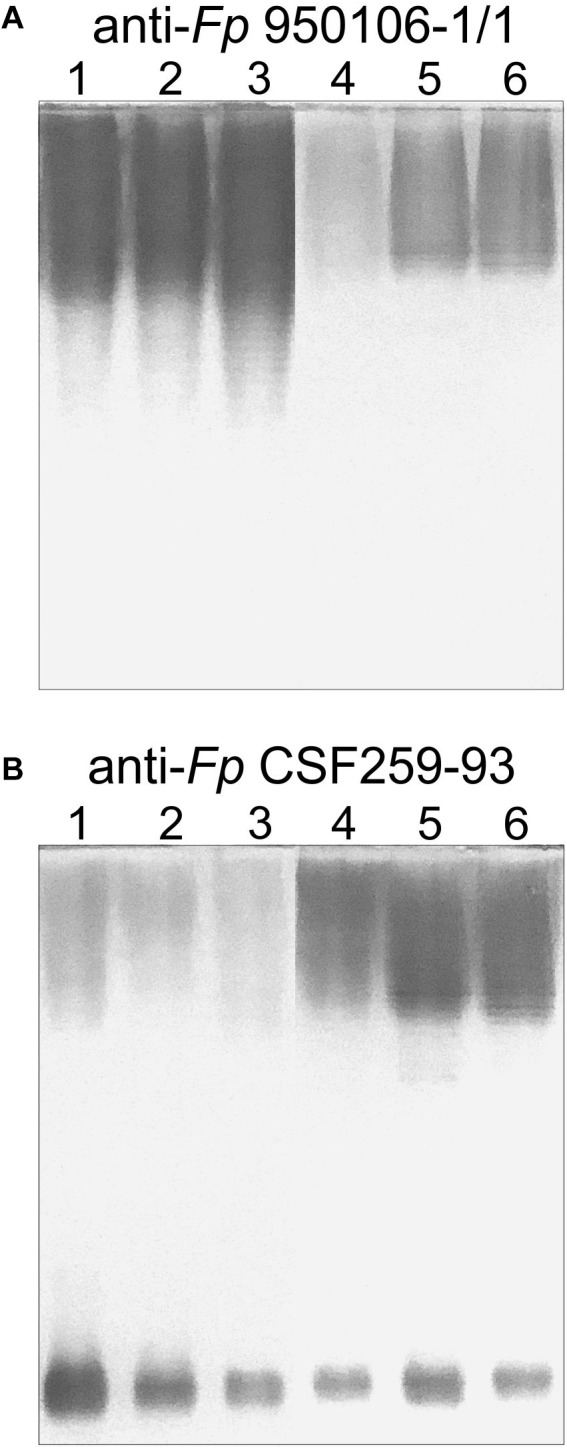
Western blots of LPS (0.5 μg/lane) developed with equivalent dilutions of **(A)** anti-*Fp* 950106-1/1 serum or **(B)** anti-*Fp* CSF259-93 serum. Number designations for LPS of different *Fp* strains are the same as in Figure 2: (1) *Fp* 950106-1/1; (2) *Fp* 11754; (3) *Fp* CSF117-10; (4) *Fp* ARS-060-14; (5) *Fp* Loa-10; and (6) *Fp* CSF259-93.

Obvious differences in the immunoreactivity of LPS from different strains were also noted on Western transfers of LPS overlaid with equivalent dilutions of rabbit antiserum against *Fp* 960106-1/1 or *Fp* CSF259-93 (Figure 3). Thus, labeling of LPS on a transfer incubated with rabbit anti-*Fp* 950106-1/1 antiserum diluted 1/16,000 was much stronger for *Fp* 950106-1/1 and other strains that carried *wzy*_1_ (Figure 3A, lanes 1, 2 and 3) than for strains that carried *wzy*_2_ (lanes 4, 5 and 6). Reduced labeling of LPS due to differences in R-group genes (Figure 2D) was not seen among the former strains (compare lane 3 to lanes 1 and 2) but was seen among the later strains (compare lane 4 to lanes 5 and 6). Compared with Figure 3A, the opposite pattern of labeling appeared on an identical transfer overlaid with anti-*Fp* CSF259-93 serum diluted 1/400 (Figure 3B). This antiserum, in addition to labeling high molecular weight LPS, labeled a rapidly migrating band (Figure 3B) in the region expected for core-Lipid A (MacLachlan et al., 1993). Weak labeling of a band in the same region, although not present in Figure 3A, was noted on an identical transfer overlaid with a 1/400 dilution of rabbit anti-*Fp* 950106-1/1 antiserum (Supplementary Figure S4).

The importance of *wzy* as a genetic determinant of both rabbit and rainbow trout antibody specificity was evident from results of ELISA inhibition experiments performed with LPS from different strains (Table 3). Thus, concentrations of homologous LPS (i.e., LPS from either *Fp* 950106-1/1 and *Fp* 11754 or *Fp* CSF259-93 and *Fp* Loa-10) for 50% inhibition of either rabbit or rainbow trout antibody binding were in the 1–10 ng/ml range whereas concentrations of heterologous LPS (i.e., LPS from strains that differed only in *wzy*) for comparable inhibition were in the 2000–3000 ng/ml range for inhibition of rabbit antibody binding and greater than 50,000 ng/ml for inhibition of trout antibody binding (Table 3). In contrast with *wzy*, the contribution of R-group genes to immunological specificity varied between antisera. Thus, low concentrations of *Fp* CSF117-10 LPS (number 3 in Table 3 and Figure 2D) completely inhibited rainbow trout anti-*Fp* 950106-1/1 antibody binding whereas this LPS at concentrations that ranged from 10 to 5000 ng/ml only partially inhibited (approximately 60%) rabbit anti-*Fp* 950106-1/1 antibody binding. Similarly, intermediate concentrations of *Fp* ARS-060-10 LPS (number 4 in Table 3 and Figure 2D) completely inhibited rabbit anti-*Fp* CSF259-93 antibody binding while this LPS at concentrations that ranged from 10 to 50,000 ng/ml only partially inhibited (approximately 40%) rainbow trout anti-*Fp* CSF259-93 antibody binding (Table 3). Thus, the association of LPS antigenicity with R-group genes was evident from reactions of rainbow trout as well as rabbit antibodies.

**Table 3:** ELISA Inhibition of rabbit and rainbow trout anti-*Fp* antibody binding to homologous LPS by LPS of different *Fp* strains.

LPS Inhibitor (*Fp* strain)	Soluble LPS (ng/ml) for 50% Inhibition of ELISA (mean ± Standard error)			
	Anti-*Fp* 950106-1/1		Anti-*Fp* CSF259-93	
	Rabbit	Trout	Rabbit	Trout
(1) 950106-1/1	0.65 ± 0.04	4.8 ± 1.8	2,200 ± 590	>50,000
(2) 11754	0.82 ± 0.06	10.3 ± 1.2	1,700 ± 270	>50,000
(3) CSF117-10	P.I. (60%)^∗^	2.4 ± 0.6	3,870 ± 770	>50,000
(4) ARS-060-14	1,000 ± 180	>50,000	825 ± 70	P.I. (40%)^∗^
(5) Loa-10	2,130 ± 150	>50,000	3.1 ± 0.6	4.9 ± 1.0
(6) CSF259-93	3,350 ± 1,100	>50,000	1.8 ± 0.2	2.3 ± 0.1

*^∗^P.I., partial Inhibition (percent) of ELISA by heterologous LPS.*

## Discussion

**FIGURE 4:**
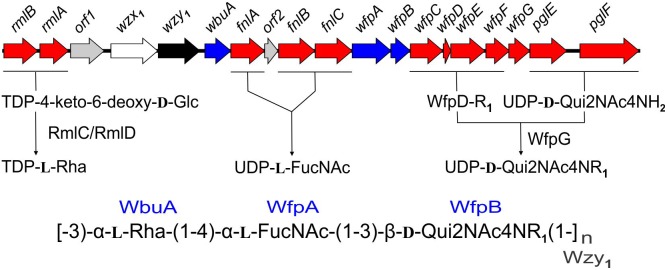
Proposed genetic basis of *Fp* 950106-1/1 O-PS structure showing genes for nucleotide sugar biosynthesis (red), glycosyl transferases (blue), polymerase (black), flippase (white), and genes of unknown function (gray), which are given *orf* designations.

The present findings provide a framework for defining the genetic basis of O-PS structure and antigenicity. To facilitate discussion, gene names have been assigned to the 19 ORFs in the O-PS gene cluster of *Fp* 950106-1/1 (Figure 4) in accordance with recommendations of the Bacterial Polysaccharide Gene Database (Reeves et al., 1996), using established designations for certain pathway genes and *wfp^∗^* designations for O-PS specific genes. That the O-PS structures of *Fp* 950106-1/1 and *Fp* CSF259-93 are identical except for the linkage of D-Qui2NAc4NR to L-Rha (Figure 1C) supports the presence of different O-antigen polymerases (i.e., Wzy_1_ and Wzy_2_) in serotype Fd and Th strains of *F. psychrophilum* (Rochat et al., 2017). Studies to genetically alter O-PS structure are underway to further establish the identity of *wzy* and obtain isogenic strains for studies of pathogenesis and immune protection. The presence of D-Qui2NAc4NR_1_ at the reducing end of putative O-PS biological repeating unit (Figure 4) indicates that the transfer of this residue to carrier lipid is the initial step in O-PS synthesis. This transfer can be attributed to *wfpB*, which encodes a homolog of PglC (Supplementary Table S1), the N,N′-diacetylbacillosamine-phosphotransferase of *C. jejuni* (Glover et al., 2006). Interestingly, the corresponding genes of *Fp* CSF117-10 and *Fp* ARS-060-14 (i.e., those designated *wfpB*) encode proteins that are virtually identical to WfpB of *Fp* 950106-1/1 except for the presence of distinct C-terminal sequences. Further studies are needed to determine whether WfpB of each former strain is tailored to a specific R-group (i.e., R_2_ or R_3_, respectively) in D-Qui2NAc4NR. The two remaining *Fp* 950106-1/1 genes for glycosyltransferases encode homologs of *Escherichia coli* O26 WbuA and WbuB (D’Souza et al., 2002). The WbuB homolog of *F. psychrophilum* has a different acceptor than WbuB of *E. coli* (i.e., D-Qui2NAc4NR vs. D-GlcNAc) and thus, has been designated WfpA in Figure 4. The WbuA designation has been retained, however, for the α-L-Rha transferase of *F. psychrophilum* as it has the same donor and acceptor as WbuA of *E. coli*. Following the WbuA-mediated transfer of α-L-Rha in *F. psychrophilum*, the lipid-linked trisaccharide is presumably flipped to the outer surface of the cytoplasmic membrane for β1-3 polymerization by Wzy_1_ of *Fp* 950106-1/1 (Figure 4) or α1-2 polymerization by Wzy_2_ of *Fp* CSF259-93.

The O-PS gene clusters of *Fp* 950106-1/1 (Figure 4) and *Fp* CSF259-93 share several genes for synthesis of nucleotide sugar precursors, including two of the four *rml* genes for conversion of α-D-Glc 1-phosphate to TDP-L-Rha (Samuel and Reeves, 2003); the other two *rml* genes (i.e., *rmlC* and *rmlD*) occur elsewhere in the chromosome. The O-PS gene cluster of *Fp* 950106-1/1 also contains three *fln* genes for conversion of UDP-D-GlcNAc to UDP-L-FucNAc and *pglE* and *pglF* for conversion of UDP-D-GlcNAc to UDP-D-Qui2NAc4NH_2_ (Kneidinger et al., 2003; Figure 4). In *C. jejuni*, which carries *pglE* and *pglF*, the additional presence of *pglD* for an acetyltransferase that N-acetylates UDP-D-Qui2NAc4NH_2_ results in formation of UDP-D-Qui2NAc4NAc (i.e., N,N′-diacetylbacillosamine) (Olivier et al., 2006). Similarly, the *pglD*-homolog designated *wfpG* in *F. psychrophilum* (Supplementary Table S1) encodes an acyltransferase that is predicted to transfer the R_1_ group from a putative O-PS-specific, acyl carrier protein encoded by *wfpD* to UDP-D-Qui2NAc4NH_2_ to form UDP-D-Qui2NAc4NR_1_ (Figure 4). The presence of an encoded acyl carrier protein clearly suggests synthesis of the R_1_-group (i.e., 3,5-dihydroxyhexanoyl) by a pathway that resembles type II fatty acid synthesis (Rock and Jackowski, 2002). Additional support for this possibility comes from the presence of adjacent *wfpC* and *wfpE* for different 3-ketoacyl-ACP synthases and *wfpF* for a 3-ketoacyl-ACP reductase (Supplementary Table S1). *Fp* CSF117-10 and *Fp* ARS-060-14 both lack genes for synthesis of the R_1_-group but each has a *pglD* homolog (*wfpH* or *wfpI* in Figure 2D and Supplementary Table S1) for an acyltransferase that is predicted to N-acylate UDP-D-Qui2NAc4NH_2_ to form UDP-D-Qui2NAc4NR_2_ or UDP-D-Qui2NAc4NR_3_, respectively. Structural characterization of O-PS from these strains is underway to identify the R-group in each polysaccharide.

Following phenol-water extraction, we recovered *F. psychrophilum* LPS from the phenol phase whereas MacLean et al. (2001) recovered *F. psychrophilum* LPS from the clear phenol-water solution obtained by dilution of 50% hot phenol. Regardless of this difference, O-PS isolated in the present study has the same structure as that proposed previously. It is important to note, however, that while the presently determined structure is based solely on glycolyl composition (Supplementary Figure S1) and NMR data (Figure 1, Supplementary Figures S2, S3, and Table 2), the structure of MacLean et al. (2001) was derived from extensive characterization of the 3,5 dihydroxyhexanoyl R-group using HF solvolysis, methanolysis, chemical synthesis, mild acid hydrolysis, mass spectrometry and NMR spectroscopy and optical rotation to determine the absolute configuration of each sugar residue and the chiral centers in the 3,5 dihydroxy R-group. Comparable studies of *Fp* 950106-1/1 O-PS were not performed in the present study. We assume, however, that the structure of the R-group and the absolute configuration of sugar residues in this polysaccharide are the same as in *Fp* CSF259-93 O-PS since the O-PS loci of these strains are the same except for *wzy*.

The association of *F. psychrophilum* serotypes Fd and Th with the presence of *wzy*_1_ or *wzy*_2_, respectively, in the O-PS gene clusters of different strains is based on the use of serotyping reagents prepared against presently characterized *Fp* 950106-1, which is serotype Fd, and *Fp* DK002, which is serotype Th (Rochat et al., 2017). Importantly, the O-PS gene cluster of the later strain is identical to that of *Fp* CSF259-93. Based on the present findings, cross adsorption of antiserum against one strain with cells of the other is expected to remove antibodies against common epitopes, including those associated with R_1_-groups, and leave antibodies against specific epitopes associated with the *wzy*-dependent linkages that distinguish each O-PS. The present findings (Figure 2, 3 and Table 3) not only support the importance of *wzy*_1_ and *wzy*_2_ as genetic determinants of serological specificity but also show the influence of R-group genes. Thus, while antigenic identity was noted between LPS from strains with identical O-PS gene clusters, non-identity was seen between LPS from strains that differed in R-group genes (Figure 2B,C). The contribution of R-group genes to differences in LPS antigenicity was also evident in ELISA inhibition experiments performed with both rabbit antisera and with one of two pooled rainbow trout antisera (Table 3). Considered together, these findings indicate that the reactions of anti-Fd and anti-Th typing sera are serogroup (rather than serotype) specific and suggest that each genetically distinct O-PS locus is associated with a different LPS serotype. The latter suggestion is not without precedent. Indeed, the validity of genetically defined serotypes is well established from studies of several other pathogens, most notably the pneumococcus, where each of the over 90 capsular polysaccharide (CPS) serotypes identified by factor antisera (Henrichsen, 1995) has been associated with a distinct *cps* locus (Bentley et al., 2006). In the case of *F. psychrophilum*, comparison of O-PS loci from approximately 60 whole genome sequences suggests 17 different LPS serotypes, 15 of which have been described (Rochat et al., 2017).

The present and previous findings (Rochat et al., 2017) provide a firm basis for development of a comprehensive genetic scheme for serotyping *F. psychrophilum* based on serogroups that differ in *wzy* and serotypes on other genes that influence O-PS structure, such as those for R-group synthesis. Development of such a scheme would not reduce the need for further immunological studies, but instead would serve to focus such studies on antigenic characterization and comparison of genetically defined LPS serotypes as well as on the ability of different serotypes to afford cross protection in vaccine studies or cause disease in studies of host genetic resistance. Widely available PCR-based methods for accurate and comparable genetic serotyping of *F. psychrophilum* would also contribute to improved disease surveillance and thereby facilitate identification and tracking of virulent serotypes as well as novel serotypes that might arise by recombination during outbreaks of disease.

## Ethics Statement

Animal studies were performed at Pacific Immunology following an approved animal protocol or at the NCCCWA following Institutional Animal Care and Use Committee (Leetown, WV, United States) protocols #98 and #139.

## Author Contributions

JC and GW designed the study. JC isolated the LPS and O-PS, performed the immunological studies, and interpreted the genetic data. CB performed the NMR studies. GW was responsible for DNA sequencing and preparation of antisera. JC and CB drafted the manuscript. All authors edited and approved the final manuscript.

## Conflict of Interest Statement

The authors declare that the research was conducted in the absence of any commercial or financial relationships that could be construed as a potential conflict of interest.

## Abbreviations

DQF-COSY: double quantum filtered coherence spectroscopy
*Fp*: *Flavobacterium psychrophilum*
HIC: hydrophobic interaction chromatography
HMBC: heteronuclear multiple bond correlation
HSQC: heteronuclear single quantum coherence
HSQMBC: heteronuclear single quantum multiple bond correlation
LPS: lipopolysaccharide
NMR: nuclear magnetic resonance
NOESY: nuclear Overhauser effect spectroscopy
O-PS: *O*-polysaccharide
TOCSY: total coherence spectroscopy.
