# Supplementary material for: Comparative Structural and Antigenic Characterization of Genetically Distinct Flavobacterium psychrophilum O-Polysaccharides
Source: Front Microbiol. 2019 May 8;10:1041. doi: 10.3389/fmicb.2019.01041 (PMC6519341; doi:10.3389/fmicb.2019.01041)
Supplement: Supplementary file 1 [file Data_Sheet_1.PDF]

Table S1. Selected homologues of *F. psychrophilum* genes for O-PS biosynthesis

Gene	Protein* (a.a.)	Selected Homologue (Accession no.)	% Identity (a.a.)	Proposed Function
rmlA	293	RmlA, *E. coli* (NP_416543.1)	66	dTDP-D-Glc-1-phosphate thymidylyltransferase
rmlB	348	RmlB, *E. coli* (NP_416545.1)	56	dTDP-D-Glc 4,6-dehydratase
fnlA	347	WbjB, *P. aeruginosa* (AAD45265)	68	UDP-D-GlcNAc 4,6-dehydratase/5-epimerase/3-epimerase
fnlB	372	WbjC, *P. aeruginosa* (AAD45266.1)	31	UDP-2-acetamido-2,6-dideoxy-β-L-lyxo-4-hexulose 4-reductase
fnlC	378	WbjD, *P. aeruginosa* (AAD45267)	65	UDP-2-acetamido-2,6-dideoxy-β-L-talose 2-epimerase
pglE	379	PglE, *C. jejuni* (CAL35238.1)	43	UDP-4-keto-6-deoxy-GlcNAc aminotransferase
pglF	608	PglF, *C. jejuni* (CAL35237.1)	36	UDP-GlcNAc C4,6 dehydratase
wbuA	266	WbuA, *E. coli* (YP_001296183.1)	48	CAZY GT2, L-Rha transferase, 1-4 transfer of α-L-Rha to α-L-FucNAc
wfpA	400	WbuB, *E. coli* (YP_002344517.1)	27	CAZY GT4, L-FucNAc transferase, 1-3 transfer of α-L-FucNAc to D-Qui2NAc4NR
wfpB	200	PglC, *C. jejuni* (YP_002344517.1)	57	Glycosylphosphotransferase, transfer of α-D-Qui2NAc4NR$_1$-phosphate to carrier lipid
wfpC	333	FabH, *B. thuingiensis* (YP_036004.1)	31	Ketoacyl-ACP synthase III, R$_1$-group synthesis
wfpD	76	AcpP, *B. uniformis* (WP_117964461.1)	39	Acyl carrier protein (ACP), carrier protein for R$_1$-group synthesis (R$_1$-ACP)
wfpE	350	FabH, *B. thuingiensis* (YP_036004.1)	31	Ketoacyl-ACP synthase III, R$_1$-group synthesis
		WfpC, *F. psychrophilum*	33	
wfpF	242	FabG, *E. coli* (NP_389732.1)	25	3-ketoacyl-ACP reductase, R$_1$-group synthesis
wfpG	216	PglD, *C. jejuni* (CAL35240.1)	34	Acyltransferase, transfer of R$_1$-group from ACP to UDP-Qui2NAc4NH$_2$
wfpH	213	PglD, *C. jejuni* (CAL35240.1)	27	Acyltransferase, transfer of R$_2$-group to UDP-Qui2NAc4NH$_2$
		WfpG, *F. psychrophilum*	32	
wfpI	205	PglD, *C. jejuni* (CAL35240.1)	35	Acyltransferase, transfer of R$_3$-group to UDP-Qui2NAc4NH$_2$
		WfpG, *F. psychrophilum*	27	
		WfpH, *F. psychrophilum*	35	

* Proteins from translated genes of *Fp* 950106-1/1 except for *wfpH* of *Fp* CSF117-10 and *wfpI* of *Fp* ARS-060-14.
