# Supplementary material for: Comparative Structural and Antigenic Characterization of Genetically Distinct Flavobacterium psychrophilum O-Polysaccharides
Source: Front Microbiol. 2019 May 8;10:1041. doi: 10.3389/fmicb.2019.01041 (PMC6519341; doi:10.3389/fmicb.2019.01041)
Supplement: Supplementary file 2 [file Data_Sheet_2.PDF]

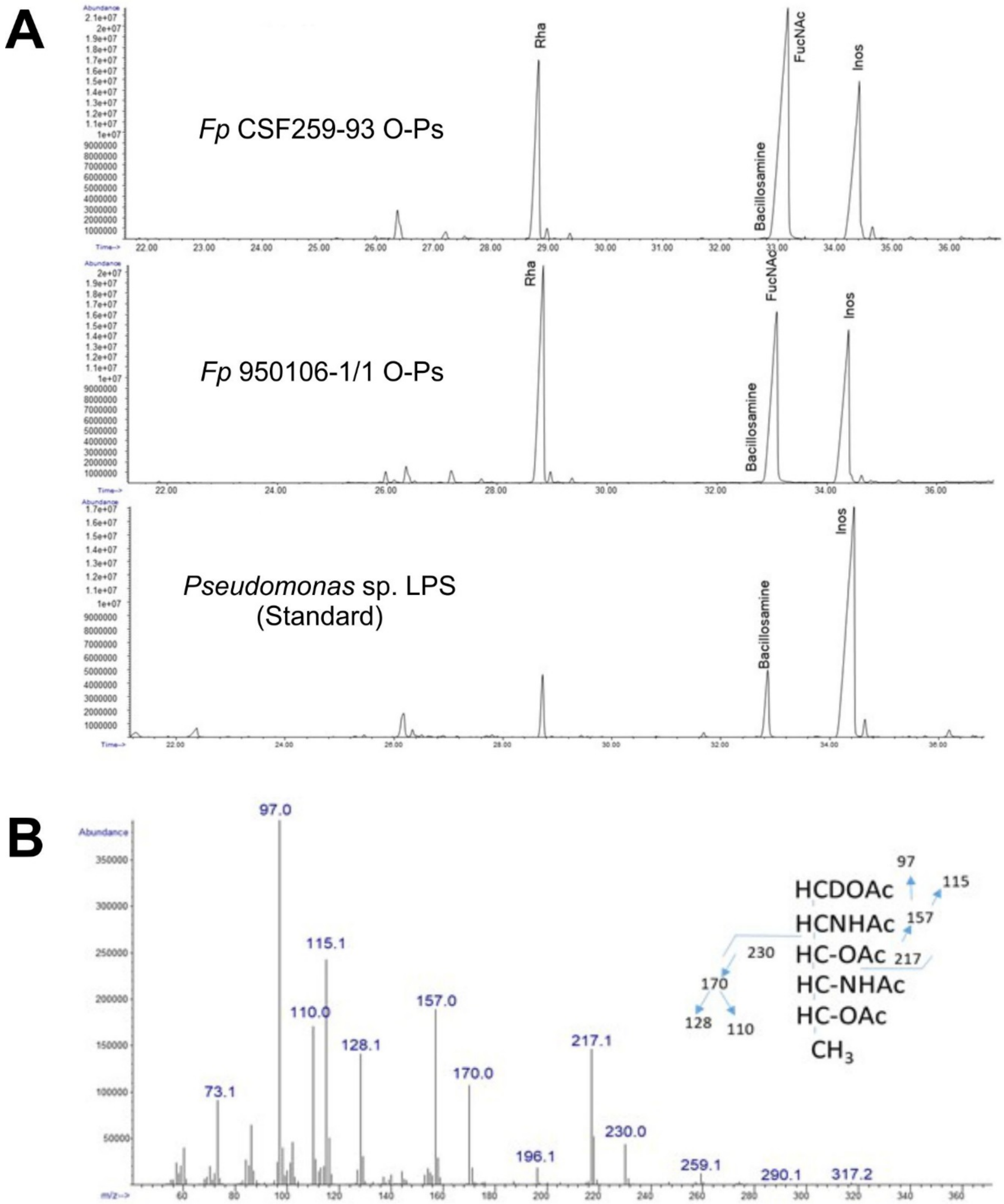

Figure S1. Glycosyl composition analysis performed by gas chromatography/mass spectroscopy (GC/MS) of alditol acetates with inositol (Inos) as standard: (**A**) chromatograms of *Fp* CSF259-93 O-PS, *Fp* 950106-1/1 O-PS and *Pseudomonas* sp. LPS (standard) showing prominent peaks of rhamnose (Rha) and N-acetylfucosamine (FucNAc) and a very small peak of bacillosamine from each O-PS sample; (**B**) mass spectrum of bacillosamine (alditol acetate) seen from each *Fp* O-PS and *Pseudomonas* sp. LPS.
