# Supplementary material for: Comparative Structural and Antigenic Characterization of Genetically Distinct Flavobacterium psychrophilum O-Polysaccharides
Source: Front Microbiol. 2019 May 8;10:1041. doi: 10.3389/fmicb.2019.01041 (PMC6519341; doi:10.3389/fmicb.2019.01041)
Supplement: Supplementary file 3 [file Data_Sheet_3.PDF]

HSQC

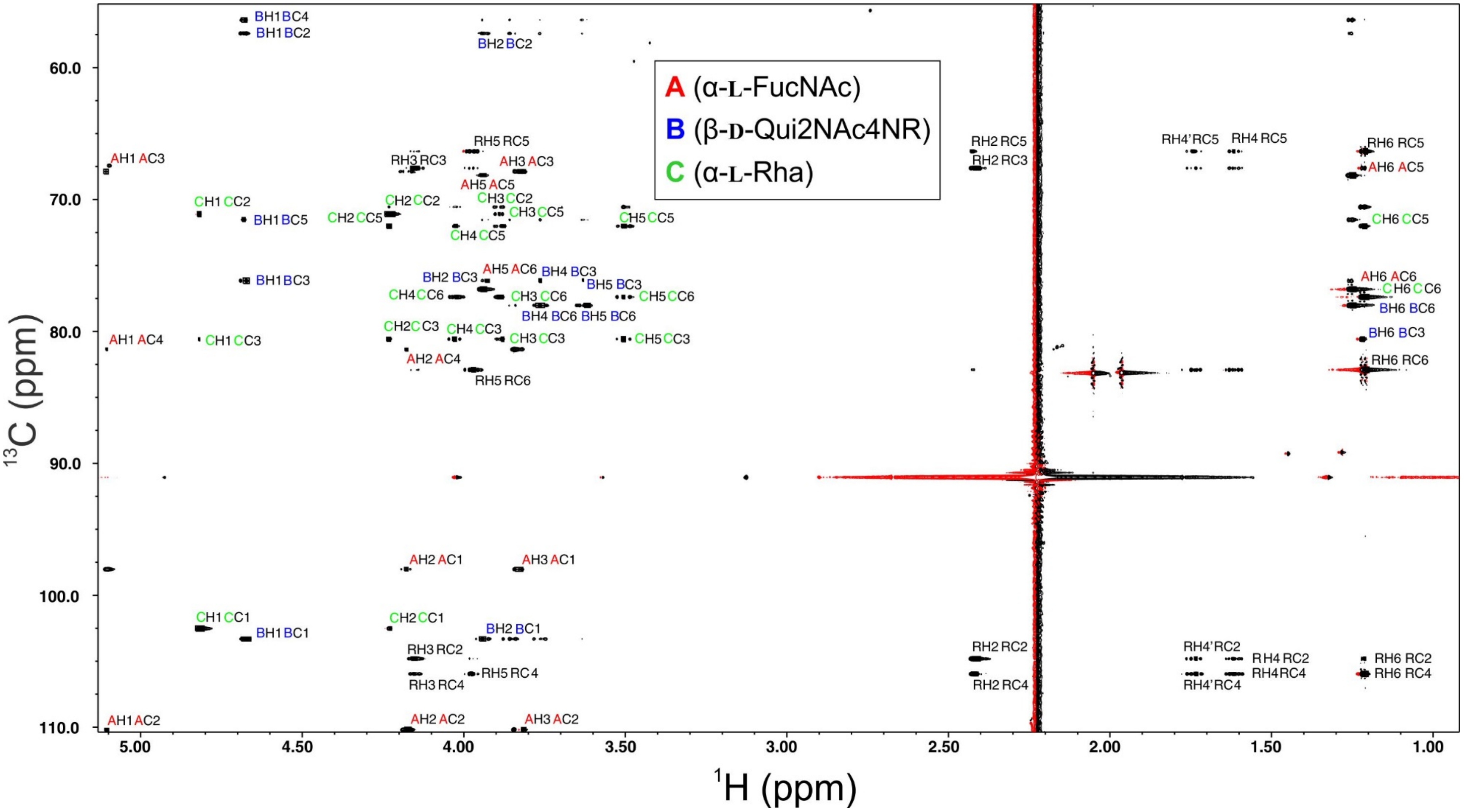

Figure S2. HSQC-TOCSY spectrum of *Fp* 950106-1/1 O-PS. Note that the ^{13}C chemical shifts for the 6-deoxy sugar methyl groups are aliased by the ^{13}C sweep width and are 60 ppm higher than the correct values given in Table 2.
