# Supplementary figures and images for: Comparative Structural and Antigenic Characterization of Genetically Distinct Flavobacterium psychrophilum O-Polysaccharides
Source: Front Microbiol. 2019 May 8;10:1041. doi: 10.3389/fmicb.2019.01041 (PMC6519341; doi:10.3389/fmicb.2019.01041)

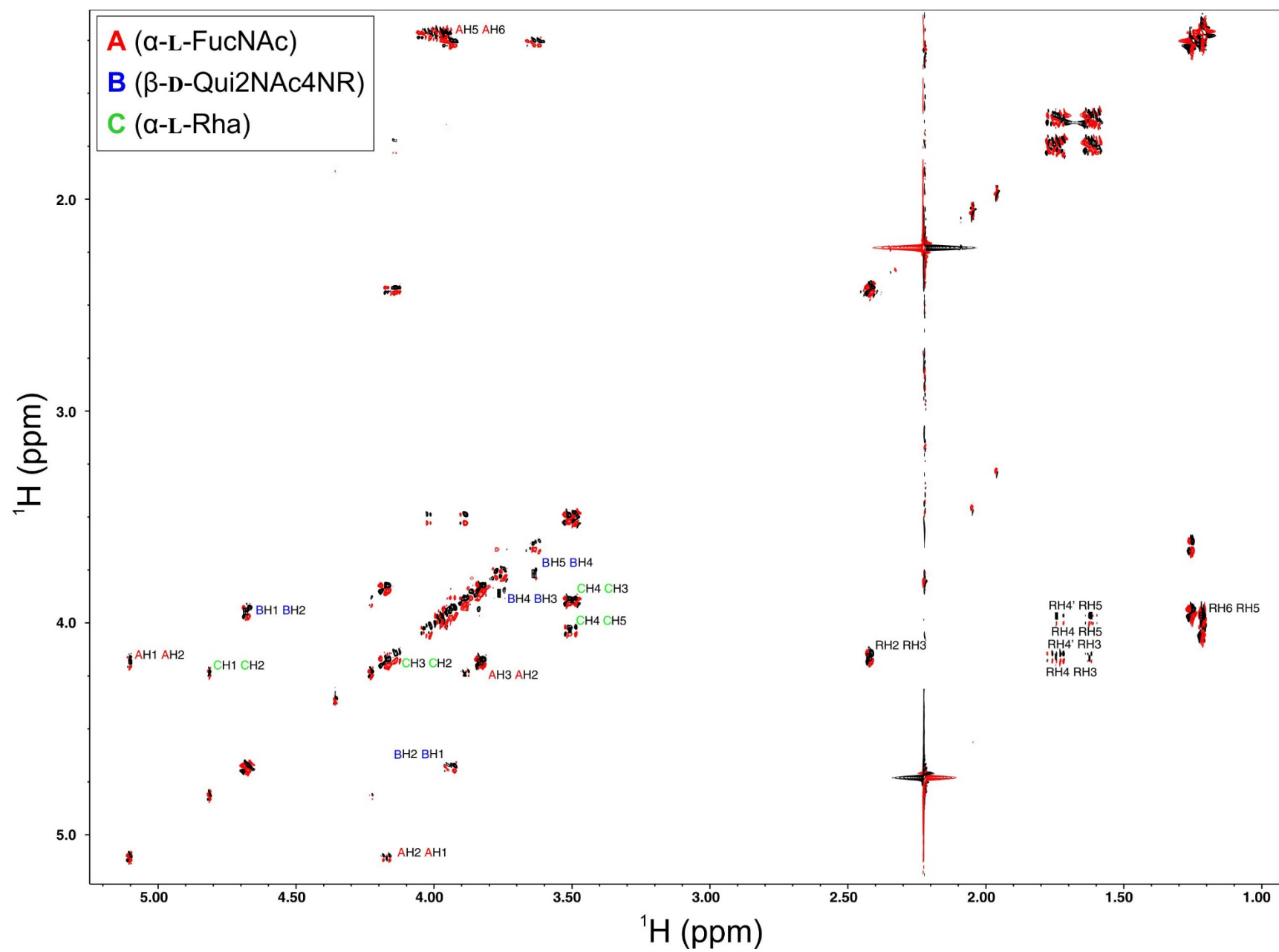

Figure S3. DQF-COSY spectrum of *Fp* 950106-1/1 O-PS.

Supplement: Supplementary file 4 [file Data_Sheet_4.PDF]
