# Supplementary material for: Comparative Structural and Antigenic Characterization of Genetically Distinct Flavobacterium psychrophilum O-Polysaccharides
Source: Front Microbiol. 2019 May 8;10:1041. doi: 10.3389/fmicb.2019.01041 (PMC6519341; doi:10.3389/fmicb.2019.01041)
Supplement: Supplementary file 5 [file Data_Sheet_5.PDF]

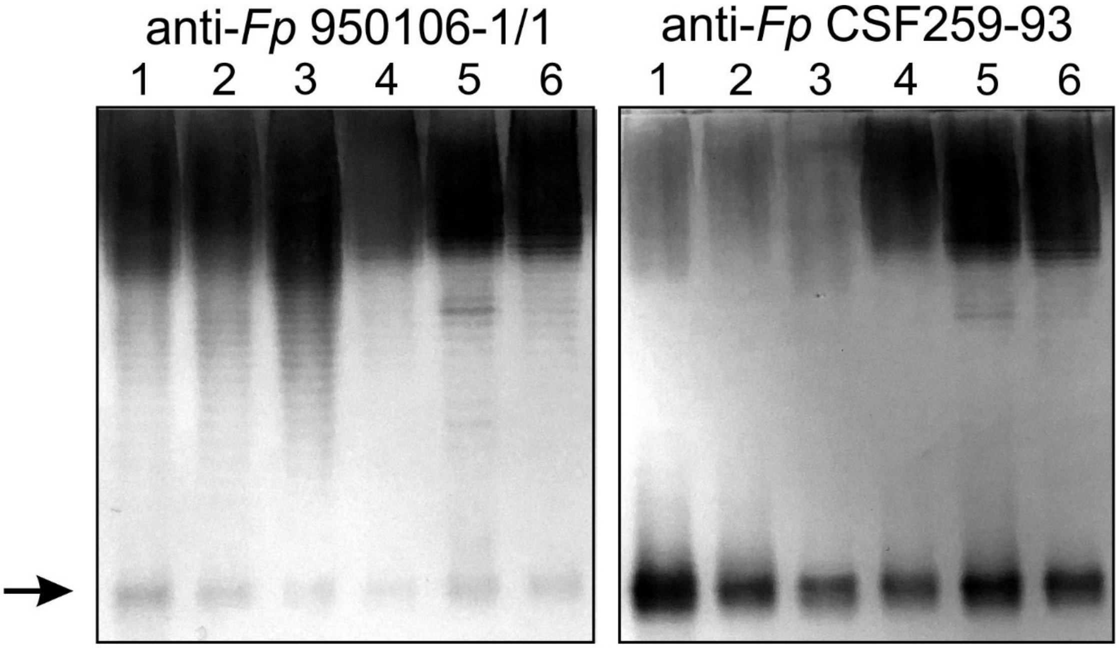

Figure S4. Western blots of LPS (0.5 μg/lane) from (1) *Fp* 950106-1/1, (2) *Fp* 11754, (3) *Fp* CSF117-10, (4) *Fp* ARS-060-14, (5) *Fp* Loa-10 and (6) *Fp* CSF259-93 developed with 1/400 dilutions of anti-*Fp* 950106-1/1 serum or anti-*Fp* CSF259-93 serum showing labeling of core-Lipid A (arrow) by both antisera.
